# Laparoscopic cholecystectomy for cholelithiasis in children

**DOI:** 10.4103/0971-9261.59602

**Published:** 2009

**Authors:** Deepak Javare Gowda, Prakash Agarwal, RajKishore Bagdi, Balagopal Subramanian, Manoj Kumar, Madhu Ramasundaram, Balamourougane Paramasamy, Zaffer Saleem Khanday

**Affiliations:** Department of Pediatric Surgery, Sri Ramachandra Medical College, Porur, Chennai – 600 116, India

**Keywords:** Children, cholelithiasis, laparoscopic cholecystectomy.

## Abstract

**Aim:**

To evaluate the role of laparoscopic cholecystectomy (LC) in the management of cholelithiasis in children.

**Methods:**

A retrospective review of our experience with LC for cholelithiasis at our institution, between April 2006 and November 2008, was done. Data included patient demographics, clinical history, hematological investigations, imaging studies, operative technique, postoperative complications, postoperative recovery, and final histopathological diagnosis.

**Results:**

During the study period of 32 months, 18 children (8 males and 10 females) with cholelithiasis were treated by LC. The mean age was 9.4 years (range 3–18). Seventeen children had symptoms of biliary tract disease and 1 child had incidentally detected cholelithiasis during an ultrasonography of abdomen for unrelated cause. Only 5 (27.8%) children had definitive etiological risk factors for cholelithiasis and the remaining 13 (75.2%) cases were idiopathic. Sixteen cases had pigmented gallstones and 2 had cholesterol gallstones. All the 18 patients underwent LC, 17 elective, and 1 emergency LC. The mean operative duration was 74.2 min (range 50–180). Postoperative complications occurred in 2 (11.1%) patients. The average duration of hospital stay was 4.1 days (range 3–6).

**Conclusion:**

Laparoscopic chloecystectomy is a safe and efficacious treatment for pediatric cholelithiasis. The cause for increased incidence of pediatric gallstones and their natural history needs to be further evaluated.

## INTRODUCTION

Cholelithiasis, although increasing in frequency in children, is still far less common than in the adult population.[1] In a population-based study, prevalence of gallstones in children was 1.9%.[2] The nature of disease process is different as compared to adults, with a higher proportion of pigment stones and less cholesterol-based stone disease in the pediatric population, especially in those younger than 10 years.[3] Laparoscopic cholecystectomy (LC) is considered to be the “gold standard” surgical procedure for cholelithiasis in adults, with vast amount of published data supporting this. However there is a paucity of reports in the literature pertaining to the clinicopathological characteristics and laparoscopic management of gallstones in children.

## MATERIALS AND METHODS

A retrospective review of all the children who underwent LC for cholelithiasis in our institution between April 2006 and November 2008 was done. The patient medical records were examined and the data pertaining to demographic information, clinical history, diagnosis, operative findings and operative technique, postoperative complications and recovery, and final histopathological diagnosis were obtained. LC was performed by different surgeons using the standard four ports technique. Here, 10-mm umbilical camera port was inserted by open technique in all the cases, which was also used for retrieval of gall bladder specimen. Two 5-mm working ports for surgeon were placed in the epigastrium and right midclavicular line in the hypochondrium or lumbar region. Another 5 mm port was inserted in the right anterior axillary line for grasping the fundus of the gall bladder for retraction, by the assistant surgeon. The position of the ports was adjusted (by placing them away form the site of the surgery) according to the size of the child. Intraoperative cholangiography was not deemed to be necessary in any of our patients. In pediatric population, the dissection around the Calot's triangle is easier and faster compared to adults, as the fat deposit is very minimum and peritoneal covering layer is thin allowing clear visualization of the anatomy. The patients were discharged when they were able to tolerate regular diet and were ambulatory. They were followed up in the outpatient clinic at least once after the discharge.

## RESULTS

During the study period, 18 patients underwent LC for cholelithiasis. The mean age was 9.4 years (range 3–18). Two children were less than 5 years, 10 were aged between 5 and 12 years, and 6 were adolescents. Eleven (61.1%) children had typical symptoms of biliary tract disease (right upper quadrant or epigastric pain, nausea, vomiting or food intolerance), 6 (33.3%) had fever in addition to above symptoms (calculous cholecystitis), and 1 child had asymptomatic gallstones, which was diagnosed incidentally on ultrasound examination of abdomen done for unrelated cause.

Duration of symptoms at diagnosis varied from 1 to 12 months (mean 2.9 months). Risk factors for the development of gallstones were present in 5 (27.8%) children only. Two had family history of gallstones, 2 were obese (BMI>30), and 1 child had undergone previous abdominal surgery and had received injection Ceftriaxone for 14 days. None of our patients were detected to have hemolytic disorders such as sickle cell disease, thalassemia, or hereditary spherocytosis after a complete workup. Complete hemogram, peripheral blood smear, and liver function tests were within normal limits in all the patients. All the children underwent abdominal ultrasound and were detected to have single or multiple gallstones. Ten (55.6%) children in addition had inflammatory features around the gall bladder.

Seventeen children underwent elective LC and 1 child was taken up for emergency LC after treating acute cholecystitis with intravenous antibiotics for 2 days. The mean operative duration was 74.2 min (range 50–180). Operative findings include omental or small bowel adhesions around the gall bladder (with or without edematous gallbladder) in 11 (61.1%) patients. The child who underwent emergency LC had empyema along with above-mentioned inflammatory features. Sixteen children had pigmented stones and 2 had cholesterol stones. Among the 16 children with pigmented gallstones, 2 had multiple gravel-like (<1 mm) stones. Tube drains were placed in 3 (16.6%) cases in which intraoperative bile spillage or gallbladder fossa ooze was present.

Two drains were removed within 24 h; the remaining one was kept for 96 h since significant serous fluid discharge was present during first 2 days postoperatively. The average duration of hospital stay was 4.1 days (range 3–6). Postoperative complications occurred in 2 (11.1%) patients. One child had significant prolonged serous discharge from the tube drain as mentioned above, and it resolved spontaneously. The other child who underwent emergency LC had postoperative fever for 3 days, which resolved with intravenous antibiotics.

Histopathological analysis of cholecystectomy specimens revealed chronic cholecystitis in 15 cases and 1 each of acute cholecystitis, chronic cholecystitis with focal ulceration, and acute chronic cholecystitis. Follow-up duration ranged from 3 to 35 months (average 17 months) and there were no cases of retained common bile duct stones in our study.

## DISCUSSION

Cholelithiasis is considered as an uncommon condition in children; however recent studies have documented an increasing incidence of this disorder.[2] This may be explained by increased availability and use of abdominal ultrasonogram in children. Pediatric cholelithiasis was viewed as a disease of prematurity, usually related to total parenteral nutrition. Various risk factors for cholelithiasis in children include hemolytic disorders, obesity, family history of gallstones, abdominal surgery, IgA deficiency, cystic fibrosis, therapy with ceftriaxone, and Gilbert's disease.

In our series, only 27.8% of patients had above-mentioned risk factors, and remaining 72.2% had idiopathic cholelithiasis. The incidence of idiopathic cholelithiasis in other reported series varies from 23% to 52.5%.[4] The trend of increasing incidence of non hemolytic cholelithiasis is also reflected in our series, with all of them belonging to non hemolytic cholelithiasis category. The mechanism of gallstone formation in these children is probably due to the combination of interacting processes including dehydration, transient hepatic dysfunction, dietary, inflammatory, hereditary, and endocrine influences, which affect the composition of bile.[5]

Approximately 80% of adults with gallstone are asymptomatic.[6] However in children asymptomatic gallstones are less frequent, with reported incidence of 10% and 33% in 2 different studies.[5] In our study, 5.6% children had asymptomatic gallstones. The incidence of gallstones among boys and girls is almost equal, with slightly high incidence among boys.[4] The sex ratio in our study was slightly in favor of females. LC in children differs from that in adults in various aspects. The first and foremost is the constraint of space. The importance of positioning the epigastric cannula in the left upper quadrant in small children cannot be overemphasized. Similarly the right-sided working and retracting ports should be placed in the lumbar or iliac region in younger children. Secondly, as mentioned earlier the dissection at the calot's triangle is relatively easier and quicker in children as the fat deposit is less and peritoneal covering layer is thin in children.

Routine intraoperative assessment for the common duct stones was not done in our series. Shawn *et al.* have reported that the incidence of subclinical common bile duct stones is low in children.[1] This finding has also been described in a small prospective pediatric study.[7] In the present series, as there was no evidence of common bile duct stones or altered liver function tests preoperatively in any of the patients, intraoperative cholangiogram was not done. None of the patients had evidence of residual ductal stones during the follow-up period. Hence a routine intraoperative cholangiogram is not recommended in children.

The natural history of cholelithiasis in children is not known[4], hence the treatment remains controversial. The clinical presentation, findings on ultrasound imaging, intraoperative finding and the final histopathological diagnosis of the gall bladder specimen did not correlate completely in our study. While only 6 (33.3%) patients had fever suggestive of clinical inflammation, 10 (55.6%) patients had ultrasound findings suggestive of inflammation and 11 (61.1%) had intraoperative evidence of inflammation. However all the 18 resected gallbladder specimen on histological analysis showed either acute or chronic inflammation. Since the natural history of gallstones in children is not known and histological evidence of inflammation is present in all the cases of cholelithiasis in our series, we suggest an LC for all children with cholelithiasis. A recently conducted multicenter study also reports structural alterations in the majority of gallbladders removed for cholelithiasis.[4] These authors also suggest that because of long life expectancy of children, expectant management of cholelithiasis may not be safe. However in adults where natural history is well documented, only 1–4% per year develop symptoms or complications of gallstone disease, only 10% develop symptoms in the first 5 years after diagnosis, and approximately 20% by 20 years.[8,9]

The mean operative duration for LC was 74.2 min in our study. This duration was between 70 and 80 min in other reported series.[1] The comparison of various parameters between LC and open cholecystectomy in one study reported significantly less duration of hospital stay and decreased overall cost in patients undergoing LC. The other advantages of LC such as decreased pain, avoidance of upper abdominal muscle cutting incision, quicker return to activity, and cosmetically better scar are well documented.[10]

## CONCLUSIONS

Laparoscopic cholecystectomy (LC) is confirmed to be a safe and efficacious treatment modality for pediatric cholelithiasis. The cause for increased incidence of pediatric cholelithiasis and their natural history need to be further evaluated. LC is much simpler in children compared to adult population, when it is performed by an experienced surgeon.
